# Novel aspects of biochemical assessment of bone remodeling and mineralization

**DOI:** 10.3389/fendo.2025.1702413

**Published:** 2025-11-24

**Authors:** Dorota Leszczyńska, Alicja Szatko, Agata Toboła, Katarzyna Karoń, Waldemar Misiorowski, Piotr Glinicki, Wojciech Zgliczyński

**Affiliations:** 1 Department of Endocrinology, Centre of Postgraduate Medical Education, Warszawa, Poland; 2 EndoLab Laboratory, Centre of Postgraduate Medical Education, Warszawa, Poland; 3 Doctoral School of Translational Medicine, Centre of Postgraduate Medical Education, Warszawa, Poland

**Keywords:** bone remodeling, bone turnover biomarkers, bone mineralization, immunoassay, RANK ligand, parathyroid hormone, vitamin D

## Abstract

The maintenance of skeletal integrity relies on bone remodeling, a dynamic process orchestrated by the interplay between osteoclasts, osteoblasts, and the regulatory network of osteocytes. Traditional bone turnover markers (BTM) provide a non-invasive tool to assess bone metabolic activity. However, their clinical utility is limited by a low specificity and poor reproducibility. Moreover, traditional BTM do not reflect osteocyte function, despite the central role of these cells in bone remodeling. Novel BTM, including proteins (namely sclerostin, DKK-1, RANKL/OPG, and periostin), lipids (namely sphingosine-1-phosphate), and miRNAs, offer more specific insights into the interactions between bone cells and molecular signaling within the bone microenvironment. These markers represent potential therapeutic targets, with anti-sclerostin antibodies already approved for osteoporosis treatment. Another fundamental aspect of skeletal integrity is the process of mineralization, which is tightly regulated by three hormones: parathyroid hormone (PTH), vitamin D, and fibroblast growth factor 23 (FGF-23). These hormones not only maintain systemic calcium-phosphate homeostasis but also exert direct effects on bone cells, thereby influencing bone remodeling. This narrative review summarizes the functions, commonly used analytical methods, and clinical applications of novel BTM. It also presents the mechanisms of action of these hormones on bone tissue, along with new analytical approaches for measuring vitamin D, PTH, and FGF-23. The application of “omics” techniques in bone remodeling assessment is also discussed, with an emphasis on the advantages and limitations of these approaches.

## 1 Introduction

Bone is a metabolically active living tissue that constantly undergoes remodeling, a process essential for maintaining proper skeletal function. The cellular components of the bone include osteocytes, osteoblasts and osteoclasts embedded in a mineralized bone matrix. The continuous and tightly regulated process of bone remodeling is known as *coupling*, in which bone resorption by osteoclasts precedes bone formation by osteoblasts (1).

In young adults, the remodeling rate is estimated at 5%, occurring within the structure known as the bone multicellular units (BMU), the number of which exceeds 1 million at a given moment in this age group (1–3). Remodeling is more active in trabecular than in cortical bone (3). The remodeling cycle encompasses five subsequent stages. The entire process is regulated by multiple autocrine, paracrine and endocrine factors and it is initiated by the retraction of the bone lining cells covering the bone surface, which attracts osteoclasts (1). Osteoclasts form resorption lacunae, in which acid produced by osteoclasts dissolves calcium hydroxyapatite, leading to the release of calcium into the bloodstream (1, 3). Simultaneous enzymatic degradation by osteoclasts results in release of the type I collagen fragments, which can be measured in the blood or/and in the 24-hour urine collection (4). This group, widely known as bone resorption markers, includes serum and urinary C-telopeptides of type I collagen (CTX-I), and N-telopeptides of type I collagen (NTX-I), urinary pyridinoline (PYD) and even more specific deoxypyridinoline (DPD) (5). Osteoclasts also release tartrate-resistant acid phosphatase type 5b (TRACP5b) – a non-specific hydrolase that enables osteoclasts migration and correlates with their activity (5).

The period of the intense bone resorption is succeeded by bone formation. Osteoblasts produce unmineralized extracellular matrix (ECM), consisting mainly of type I collagen, which subsequently undergoes the process of mineralization. The intensity of bone formation correlates with the blood concentrations of osteocalcin (OC), procollagen I N-propeptide (PINP), and bone-specific alkaline phosphatase (BALP), traditionally referred to as bone formation markers (5). After contributing to bone formation, osteoblasts either apoptose or differentiate into lining cells or osteocytes. Osteocytes form an extensive dendritic network, which is essential for coordinating the activities of both osteoblasts and osteoclasts (6). Scientific advances in recent years have redefined osteocytes from metabolically inactive cells to central regulators of bone cell communication. Osteocytes respond to mechanical and hormonal stimuli, which they transduce to osteoblasts and osteoclasts *via* paracrine signaling or direct cell-to-cell communication through their long cytoplasmic extensions (7). The main mediators of paracrine communication are receptor activator for nuclear factor κB ligand (RANKL), a key regulator of osteoclastogenesis, and sclerostin, a major antagonist of the wingless-related integration site/β-catenin (Wnt/β-catenin) signaling pathway. Mechanical stimuli inhibit osteocyte apoptosis and trigger the Wnt pathway, thereby promoting bone formation. In contrast, factors such as sex steroid deficiency, glucocorticoid exposure, hypoxia, aging, tumor necrosis factor alpha (TNF-α), lack of mechanical load, microdamage, fatigue, and inflammation activate pro-apoptotic pathways in osteocytes (7). This leads to the recruitment of osteoclasts and the stimulation of bone resorption. One proposed mechanism underlying this process is the upregulation of RANKL expression in osteocytes adjacent to apoptotic osteocytes (8, 9).

The unique crosstalk between bone cells and their activity is the source of numerous compounds released into the bloodstream, commonly referred as bone turnover markers (BTM), which have enabled the diagnosis and monitoring of bone diseases for over 100 years.

This narrative review begins with an overview of the limitations associated with classical BTM. Subsequently, we present a comprehensive review of novel BTM, alongside with the development of diagnostics laboratory methods used to determine their concentrations. Eventually, we discuss the future directions of assessment of bone remodeling, including “omics” techniques.

## 2 Traditional bone turnover biomarkers: advantages and disadvantages

**Figure 1:**
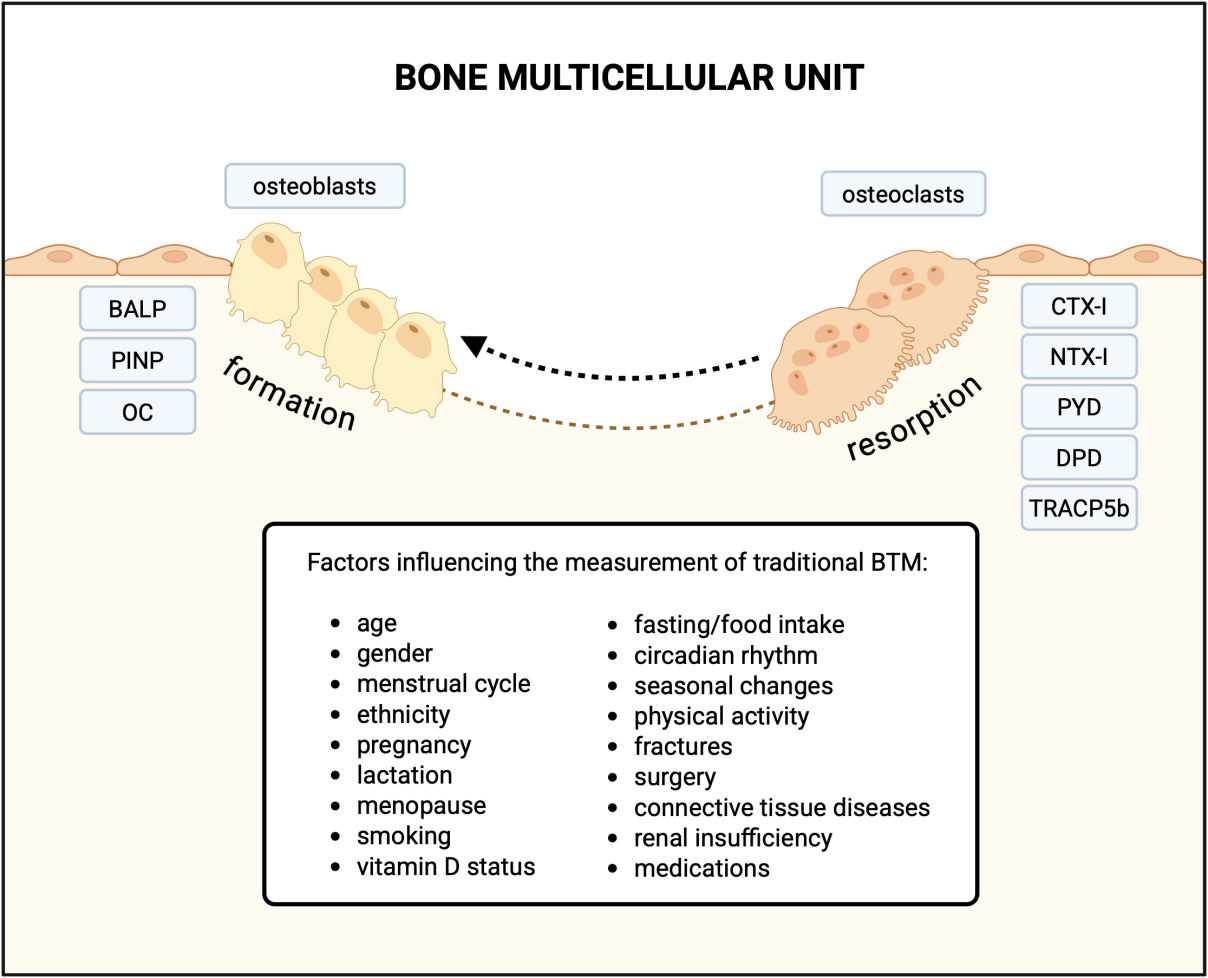
Traditional bone turnover markers associated with bone formation and resorption, and factors influencing their measurement in blood (serum/plasma) and urine. BALP, bone-specific alkaline phosphatase; PINP, procollagen I N-propeptide; OC, osteocalcin; CTX-I, C-telopeptide of type I collagen; NTX-I, N-telopeptide of type I collagen; urinary PYD, pyridinoline; DPD, deoxypyridinoline; TRACP5b-tartrate-resistant acid phosphatase type 5b; BTM, bone turnover markers. Created in BioRender.

Traditional BTM are a group of protein-based indicators that allow for non-invasive assessment of bone formation and resorption. In contrast to bone biopsy with histomorphometry, BTM reflect the remodeling activity across the entire skeleton. A further advantage of BTM is their ability to rapidly reflect changes in bone metabolic activity, in contrast to imaging techniques. However, traditional BTM have limited clinical applicability due to several limitations that may compromise their reliability and validity (10). The presence of type I collagen in other organs such as skin, tendons, and blood vessels limits the bone specificity of both resorption and formation markers derived from type I collagen metabolism, including CTX-I, NTX-I, and PINP (11, 12). Diseases affecting these tissues, including systemic sclerosis, cardiomyopathy or congestive heart failure, are associated with elevated levels of those markers (13–15). Moreover, their clinical utility is restricted by significant intra-individual, and inter-laboratory differences in reproducibility, as well as pre-analytical variability (10). The circadian rhythm, food intake, drugs, immobilization, and smoking are examples of modifiable sources of variability of BTM (4). Unmodifiable factors such as age, sex, fracture, pregnancy, lactation, and menopause should also be considered in the interpretation of the laboratory results (4). Additionally, impaired renal function may be another limiting factor. BTM such as CTX-I, NTX-I, monomeric PINP, and OC undergo renal clearance and typically accumulate in the setting of renal insufficiency (5). Factors influencing the traditional BTM are presented in **Figure 1**.

Although the discovery of BTM has broadened the spectrum of tools for assessing skeletal metabolism, their clinical utility remains limited. Most studies have shown a negative correlation between bone resorption markers and bone mineral density (BMD), and a positive correlation between concentrations of these markers and the risk of fractures in postmenopausal women (16, 17). However, not all results are consistent. A recent study led by Crandall et al., did not establish the efficacy of CTX and PINP measurements in predicting hip fracture risk in postmenopausal women (18). Currently, BTM assessments are not included in the Fracture Risk Assessment Tool (FRAX) for estimating 10-year fracture risk and are not used in the routine diagnosis of osteoporosis (19). According to osteoporosis guidelines, the clinical application of BTM is limited to evaluating responses to anabolic and antiresorptive therapies, as well as assessing patient adherence to treatment. (19). BTM, particularly PINP, are used in the clinical diagnosis of Paget’s disease and in assessment of the efficacy of the therapy (20). Although BTM are not used in the diagnosis or monitoring of primary hyperparathyroidism (PHPT), some studies have demonstrated that specific markers, such as CTX-I and PINP, would be useful in predicting changes in bone mass following successful parathyroidectomy (21, 22).

## 3 Novel bone turnover biomarkers

In recent years, growing interest in the molecular regulation of bone remodeling has led to the identification of novel signaling pathways involved in skeletal homeostasis, such as the Wnt/β-catenin pathway, the receptor activator of nuclear factor κB/receptor activator of nuclear factor κB ligand/osteoprotegerin (RANK/RANKL/OPG) system, and sphingosine-1-phosphate (S1P) signaling. A deeper understanding of these pathways has facilitated the discovery of new biomarkers that offer more specific insights into the activity of osteoblasts, osteoclasts, and osteocytes, compared to traditional BTM. These biomarkers represent a biochemically diverse group of molecules, including glycoproteins such as sclerostin and the Wnt antagonist Dickkopf-1 (DKK-1); proteins including RANKL, OPG, and periostin; lipids such as S1P; and small non-coding RNAs, including diverse group of microRNAs. Their structural heterogeneity reflects the multifaceted regulation of bone remodeling at the molecular level.

### 3.1 Protein and protein-derived bone turnover biomarkers

#### 3.1.1 Sclerostin

Sclerostin, encoded by the SOST gene and secreted mainly by mature osteocytes, is an extracellular negative regulator of Wnt/beta-catenin signaling pathway (23, 24). Since activation of this pathway stimulates bone formation, sclerostin inhibits osteogenesis by suppressing the pathway and reducing osteoblast function (23). Moreover, by upregulating osteocyte expressed RANKL, sclerostin promotes bone resorption processes (23, 24). Understanding the role of sclerostin in bone remodeling led to the development and subsequent approval of romosozumab — a humanized monoclonal anti-sclerostin antibody — for the treatment of osteoporosis in postmenopausal women at high risk of fractures, by both the Food and Drug Administration (FDA) and the European Medicines Agency (EMA) in 2019 (24). Nevertheless, the clinical utility of serum/plasma sclerostin measurement in predicting the therapeutic response to romosozumab has not yet been established (25).

##### 3.1.1.1 Immunoassays for sclerostin determination

Circulating sclerostin levels in human serum and plasma are most commonly quantified using enzyme-linked immunosorbent assays (ELISA), (e.g., Biomedica (Austria), TECOmedical (Switzerland), R&D Systems (USA)) (26, 27). Alternative methods include a multiplex electrochemiluminescence assay (Meso Scale Discovery (USA)) and a fully automated chemiluminescence immunoassay (CLIA) such as the DiaSorin LIAISON^®^ L/XL (26); however, the latter method is not currently available. Recently developed assays also allow for the quantification of bioactive (intact) sclerostin concentrations (26, 27). Importantly, the availability of multiple commercial ELISA kits from different manufacturers, with varying degrees of sensitivity and specificity, significantly limits comparability across studies and contributes to inconsistent conclusions.

##### 3.1.1.2 Clinical application

Numerous researchers have focused on evaluating the clinical relevance of sclerostin in relation to osteoporosis and its potential role in predicting fracture risk. Multiple studies have reported lower sclerostin levels in patients with osteoporosis or osteopenia compared to individuals with normal bone mass (28, 29), a finding also observed in postmenopausal women (30), which may be attributed to an age-related decline in osteocyte number (28). Gorter et al. observed that osteoporotic patients with low-energy extremity fractures exhibited lower sclerostin levels compared to non-osteoporotic fracture patients (29). These findings suggest that sclerostin may serve as a novel biomarker for osteoporosis in patients with fractures (29). On the other hand, numerous studies have reported conflicting results regarding the correlation between serum sclerostin concentrations and fracture risk (25). Moreover, research groups from China (31) and Malaysia (30) demonstrated that in women with postmenopausal osteoporosis, serum sclerostin levels were positively correlated with BMD (30, 31), and could be considered an indirect predictor of bone strength in this population (31). In the OFELY study on postmenopausal women, serum sclerostin concentrations were positively associated with bone mineral density but showed no significant relationship with the risk of incident fractures (32). The authors suggested that this discrepancy might be attributed to the fact that circulating sclerostin levels mainly reflect the number of osteocytes rather than the metabolic activity of individual cells. Since sclerostin may act predominantly at the local (bone tissue) level, peripheral concentrations may not adequately capture its paracrine effects within the bone microenvironment (32). The lack of association could also be related to the relatively small number of fracture cases and to methodological aspects, such as reliance on single morning measurements despite known diurnal variation in serum sclerostin levels (32).

Although sclerostin reflects osteocyte number and/or activity and bone remodeling processes, circulating sclerostin levels have not shown a consistent relationship with bone mineral density in either the general population or osteoporotic patients. Based on current evidence, sclerostin appears to be more informative in specific pathological conditions than as a stand-alone bone turnover marker. In a study of patients with renal osteodystrophy, osteocytic sclerostin expression was found to vary inversely with turnover rate, making it a potential marker for distinguishing between high- and low-turnover bone states in this group (23).

The assessment of sclerostin levels may offer potential benefits in the evaluation of metabolic bone disorders. Given its elevated levels in osteogenesis imperfecta (OI), (which exhibits the highest reported concentrations of sclerostin), X-linked hypophosphatemia (XLH), and Paget’s disease of bone (PDB), the assessment of circulating sclerostin may represent a useful adjunct in the diagnostic evaluation of these conditions (33). In Gaucher disease, increased sclerostin levels have been associated with skeletal manifestations, including bone pain, bone marrow infiltration, and Erlenmeyer flask deformities (34).

#### 3.1.2 Dickkopf-1

DKK-1 is a glycoprotein that, due to its mechanism of action—namely inhibition of the Wnt/β-catenin signaling pathway—shares functional similarities with sclerostin. It is primarily expressed in osteocytes and osteoblasts, as well as in the skin and placenta. In the context of bone remodeling, DKK-1 competitively binds to LRP5/6 co-receptors, thereby inhibiting Wnt-induced osteoblast differentiation and suppressing bone formation (35). Elevated DKK-1 levels have been associated with enhanced resorption, which may contribute to bone loss and altered turnover states.

##### 3.1.2.1 Immunoassays for DKK-1 determination

Commercially available ELISA kits (e.g., R&D Systems (USA), SunRedBio (China), Abcam (UK), Cloud-Clone (China)) are widely utilized in both clinical and research settings to quantify DKK-1 levels in serum or plasma. These assays offer a reliable and relatively straightforward method for monitoring DKK-1 concentrations. Recently, aptamer-based assays [oligonucleotides (short fragments of DNA or RNA) or peptides that bind specifically to a specific molecule] have emerged as a promising alternative, combining the high specificity of antibodies with the structural flexibility of aptamers, and have been validated against conventional ELISA immunoassays (36).

##### 3.1.2.2 Clinical application

DKK-1 acts as a regulatory molecule, reflecting the severity of several bone-related diseases and representing a potential therapeutic target. Elevated levels of DKK-1 have been associated with improved BMD, microarchitecture, and strength in postmenopausal women with osteoporosis (31). This paradoxical finding—similar to what is observed with sclerostin—may be explained by the hypothesis that DKK-1 levels reflect osteocyte number. Conversely, Ahmed et al. reported that postmenopausal women with significantly elevated serum DKK-1 levels exhibited more severe osteoporosis at the lumbar spine and femoral neck, suggesting that DKK-1 inhibition could hold therapeutic potential in this population (37). Additionally, an analysis by Alam et al. identified DKK-1 as part of a gene triplet associated with treatment response to bisphosphonates such as ibandronate and alendronate (38). Despite inconsistent findings regarding the overall correlation between DKK -1 concentration and BMD, the assessment of DKK- 1 levels may be particularly useful in the diagnosis and management of specific disease entities. Colditz et al. demonstrated a critical role for DKK-1 in the pathogenesis of glucocorticoid (GC)-induced bone loss (39), highlighting its potential as a therapeutic target to reduce the risk of osteoporosis resulting from long-term GC therapy. In rare bone diseases, such as osteogenesis imperfecta, which is characterized by recurrent fractures and skeletal deformities, the use of DKK-1 antisense treatment has shown promise in improving bone mechanical properties (40). In Gaucher disease, an altered sclerostin/DKK-1 ratio has been found to correlate with decreased bone mineral density, suggesting its potential utility as a biomarker of skeletal involvement (34). Moreover, elevated concentrations of DKK-1 in serum and tumor tissues of patients with various malignancies (41)—such as breast, prostate, and lung cancers—and its proposed involvement in osteolytic bone metastases support its role as a pro-tumorigenic factor, as demonstrated in both *in vivo* and *in vitro* studies (41). These findings provide a rationale for the potential use of anti-DKK-1 therapies in cancer immunotherapy (41). In multiple myeloma, increased serum DKK-1 levels correlate positively with the severity of osteolytic lesions and with treatment response, further underscoring its clinical relevance (42). Moreover, studies indicate a role for DKK-1 in the diagnosis and monitoring of chronic immunoinflammatory rheumatic diseases, which are often associated with abnormal bone remodeling, including early-stage spondyloarthritis (43). In psoriatic arthritis and ankylosing spondylitis, DKK-1 is notably elevated in axial disease forms, suggesting its utility as a biomarker for axial skeletal involvement (44).

#### 3.1.3 RANKL and osteoprotegerin

The RANK/RANKL/OPG signaling pathway regulates bone turnover by controlling the differentiation and survival of osteoclasts (45). RANKL binds to transmembrane receptor RANK on osteoclast precursors, consequently provoking their differentiation and fusion, as well as stimulating their function and survival. OPG acts as a decoy receptor for RANKL, blocking its interaction with RANK, thereby preventing osteoclast formation and inhibiting bone resorption. Both RANKL and OPG are produced by osteoblasts and osteocytes. Their expression is regulated by various stimuli such as PTH, 1,25(OH)_2_D, reproductive hormones, glucocorticosteroids, and proinflammatory cytokines.

##### 3.1.3.1 Immunoassays for RANKL and OPG determination

RANKL and OPG serum concentrations are measured using immunoassay methods such as ELISA, CLIA. However, discrepancies between findings have revealed the unsatisfactory reproducibility of RANKL and OPG measurements, which has been attributed to the lack of a standardized method and test units (46, 47). Due to these issues, new methods are being explored, including Multiplex electrochemical detection techniques (48).

Another important consideration is that available assays measure only soluble RANKL, whereas a substantial portion of RANKL remains membrane-bound on the surface of osteocytes and does not enter the circulation. The soluble form is produced through proteolytic cleavage of the membrane-bound protein (49).

RANKL/OPG ratio is a parameter that integrates both biomarkers as they function within an interdependent pathway. It has been established that the ratio demonstrates bone turnover trends more accurately than the individual concentrations of OPG and RANKL (45). However, some studies use the inverse: OPG/RANKL ratio (46, 50). Further standardization of this marker is needed.

##### 3.1.3.2 Clinical application

As key factors in regulating osteoclastogenesis, RANKL and OPG serum level measurements were initially considered promising as bone turnover markers. However, results from numerous studies have been inconsistent.

Osteoporosis has been associated with an increased RANKL/OPG ratio (46, 51, 52), low OPG (51–54), and high RANKL serum levels. Some studies have also shown a negative correlation between a high RANKL/OPG ratio and low BMD (52). Yet, findings from other research differ, reporting no significant differences in these biomarkers between osteoporotic and healthy individuals (55). Some studies have even produced contradictory results, linking high OPG levels, low RANKL levels, and a low RANKL/OPG ratio to osteoporosis (56). Reports using OPG and RANKL serum levels or the RANKL/OPG ratio to estimate the effectiveness of osteoporosis treatment have also shown discrepant results (57, 58).

#### 3.1.4 Periostin

Periostin (PSTN) is an extracellular matrix protein that participates in cortical bone metabolism and tissue healing (59). Its expression is highest in collagen-rich connective tissues, such as periosteum, periodontal ligaments, tendons, skin, aorta, and heart valves. PSTN promotes cell migration, adhesion, and proliferation by binding to integrins’ αvβ3 and αvβ5 receptors on the cell surface and activating Wnt/β-catenin, NF-κB/STAT3, PI3K/Akt, and focal adhesion kinase signaling pathways. Its elevated expression has been observed in various types of neoplasms and inflammatory diseases.

In bone, PSTN interacts with bone morphogenetic protein-1 (BMP-1), which leads to the activation of lysyl oxidase, an enzyme that catalyzes collagen cross-linking (60). This process is essential for high-strength bone formation. It has been observed that PSTN expression is increased by mechanical stress and inflammation. The protein promotes bone repair by recruiting stem cells to the injury site, enhancing osteoblast differentiation and survival, supporting matrix remodeling and mineralization. Another way in which PSTN affects osteogenesis is by downregulating sclerostin expression *via* the Wnt-β-catenin pathway.

##### 3.1.4.1 Immunoassays for PSTN determination

PSTN serum/plasma concentrations can be measured using different immunoassay methods. PSTN assays demonstrate high analytical validity and reproducibility. Standardization and reference ranges are well established, and results remain stable under normal storage conditions (61). PSTN essays are commercially available for clinical and research use. In healthy individuals, levels are high at age 16–18, then decrease and remain stable between the ages of 32 and 70, and are not influenced by gender (62).

An important limitation of PSTN assay is its low specificity, as it is expressed in various tissues, and is upregulated in numerous conditions, primarily in diseases characterized by type 2 inflammation, tissue remodeling, or fibrosis, such as chronic obstructive pulmonary disease, asthma, chronic kidney disease, diabetes, chronic heart failure, and certain types of malignancies (63). In 2017, Garnero et al. developed an ELISA for the Cathepsin K–generated periostin fragment (K-PSTN), a bone-specific PSTN form produced by osteoclastic proteolysis (64). The assay demonstrated low variability and adequate sensitivity for serum measurements in healthy individuals and was validated in postmenopausal women, showing bone specificity and correlation with cortical bone microstructure, but not with BMD, or standard bone turnover markers (65). It is not currently available for clinical use as standardized, commercially available assays and reference ranges are not established.

##### 3.1.4.2 Clinical application

High serum PSTN concentrations have been associated with postmenopausal osteoporosis, with numerous studies demonstrating a negative correlation between circulating PSTN and BMD (66, 67). However, the findings are not entirely consistent, as a few studies have failed to confirm this relationship (68, 69).

PSTN has been identified as an independent predictor of fracture risk in postmenopausal women (70, 71). In a prospective cohort study, serum K-PSTN levels were likewise associated with fracture risk, and incorporating K-PSTN into models based on BMD or FRAX significantly enhanced their diagnostic accuracy (65).

In patients with PHPT, serum PSTN levels were significantly elevated compared to healthy controls (72). Among the PHPT group, those with osteoporosis had notably higher PSTN levels than those without (72). PSTN has been identified as a predictor of osteoporosis in this population (73).

### 3.2 Lipids and lipid-derived bone turnover biomarkers

#### 3.2.1 Sphingosine 1-phosphate

Sphingosine-1-phosphate (S1P) is a bioactive sphingolipid metabolite generated by the phosphorylation of sphingosine via sphingosine kinases 1 and 2 (SK1 and SK2) (74). It acts both intracellularly and extracellularly through five distinct G protein-coupled receptors (S1PR1–S1PR5) (74–77), regulating a wide range of cellular processes including proliferation, apoptosis, and angiogenesis (74–78). In bone tissue, S1P mediates the crosstalk between osteoclasts, osteoblasts, and vascular endothelial cells, for example by recruiting osteoclast and osteoblast precursors to sites of bone injury (75), thereby coordinating bone resorption and formation (74–78). This signaling axis is increasingly recognized as a potential therapeutic target in bone-related diseases. Beyond the skeletal system, S1P receptors are expressed in multiple systems, including the immune, cardiovascular, reproductive, and nervous systems (74, 77).

##### 3.2.1.1 Immunoassays for S1P determination

The gold standard for S1P measurement remains liquid chromatography–tandem mass spectrometry (LC–MS/MS) (79), owing to its high specificity, sensitivity, and reproducibility. Both total and specific protein-bound (i.e. albumin-bound or low-density lipoprotein-bound) S1P fractions can be quantified, which may exert distinct biological effects, depending on the carrier (75, 80). However, Song et al. showed that only total plasma S1P levels correlated positively with osteoporotic fracture risk (75, 80). Recent technical improvements include the use of QTRAP^®^ LC-MS/MS technology, achieving detection limits as low as 1 nM (81). Although ELISA immunoassays are commercially available for S1P determination, they offer lower specificity compared to MS-based approaches and are more susceptible to cross-reactivity.

##### 3.2.1.2 Clinical application

Lee et al. demonstrated an association between elevated S1P levels and reduced bone strength in postmenopausal women, highlighting its potential utility in predicting fracture risk (82). Notably, S1P may serve as an independent predictor of fracture risk beyond traditional assessment tools such as FRAX (83), and incorporating S1P measurements into FRAX could enhance its clinical predictive value (84). Frost et al. proposed that S1P may act as a biomarker for the early detection of osteoporosis and could have therapeutic potential (75). For instance, S1PR3 agonists have been shown to enhance bone formation by promoting osteoblast differentiation, whereas S1PR2 antagonists may suppress bone resorption, offering targeted strategies for osteoporosis management (75). Wagner et al. reported that pharmacological elevation of S1P, *via* upregulation of S1PR3 signaling, supported bone regeneration in a model of posttraumatic osteomyelitis (85). Moreover, in Paget’s disease, S1PR3 antagonists might help mitigate excessive bone formation (86). While preclinical studies suggest that inhibition of S1PR2 or modulation of S1PRs can reduce inflammatory bone loss, their translation to human therapy is limited by potential adverse effects (87). The pro-angiogenic activity of the S1P–S1PR signaling axis, which contributes to tumor progression, including in osteosarcoma, offers a novel therapeutic avenue for targeting tumor-associated angiogenesis (88, 89). Interestingly, a negative correlation between S1P and parathyroid hormone level was found in patients with PHPT (90), although the clinical significance of this relationship requires further investigation.

**Figure 2:**
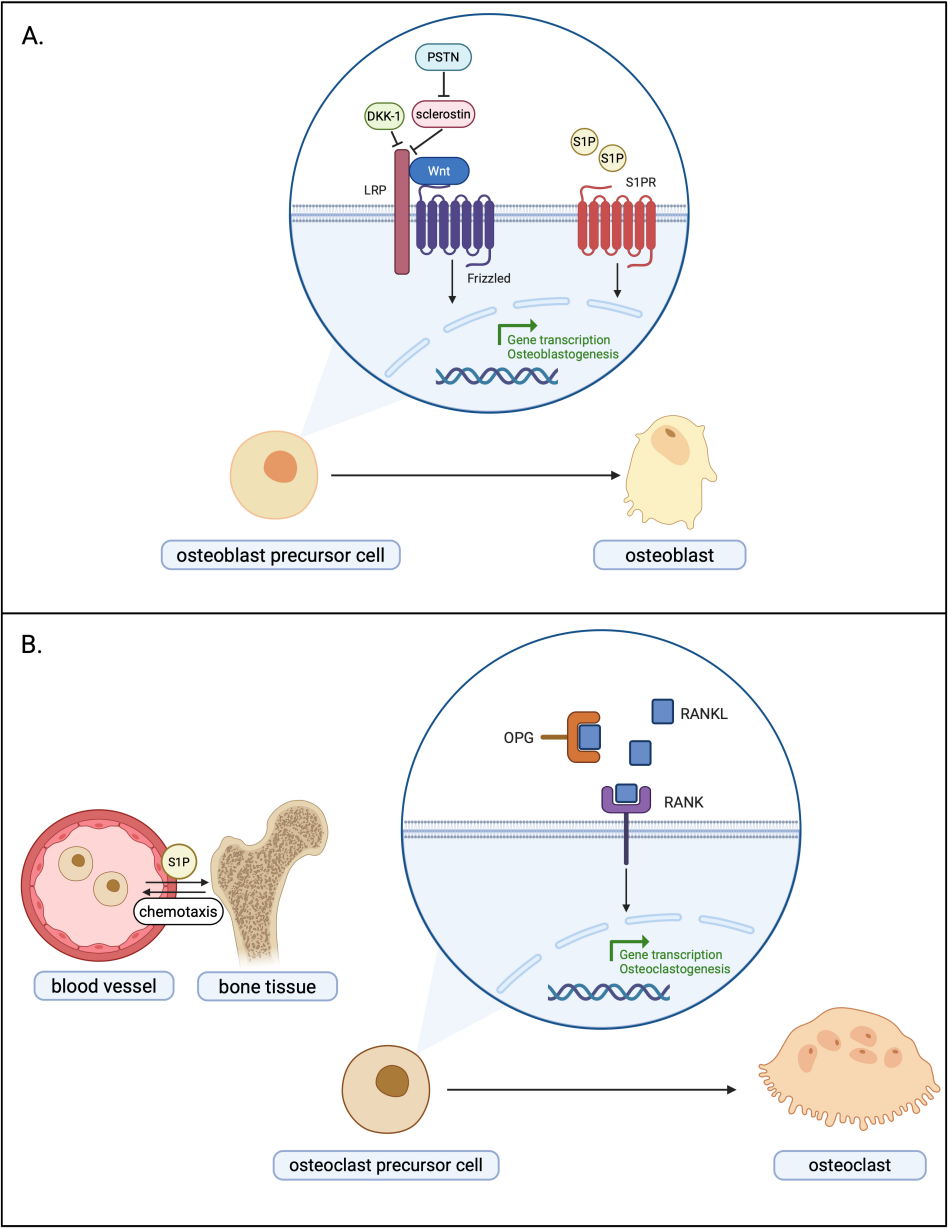
Associations of novel protein and lipid bone turnover markers with cellular pathways in osteoblast precursor cell **(A)** and osteoclast precursor cell, including the role of S1P gradient and receptor activation in regulation of osteoclast precursor cell migration between blood and bone tissue **(B)**. DKK-1,Dickkopf-1; LRP, lipoprotein receptor-related protein; PSTN – periostin; S1P, sphingosine 1-phosphate; S1PR, sphingosine 1-phosphate receptor; OPG, osteoprotegrin; RANKL, receptor activator of nuclear factor B ligand; RANK, receptor activator of nuclear factor B.Created in BioRender.

Associations of novel protein and lipid bone turnover markers with cellular pathways in bone precursor cells are presented in the **Figure 2**.

### 3.3 MicroRNA

MicroRNAs (miRNAs), small non-coding RNAs (18–22 nucleotides), secreted by numerous cells into the extracellular space, modulate gene expression post-transcriptionally and have emerged as significant regulators in bone metabolism (91, 92). Several miRNAs have been shown to modulate key signaling pathways involved in osteogenesis and osteoclastogenesis, including Wnt/β-catenin, RANK/RANKL/OPG, and bone morphogenetic protein (BMP) signaling (93, 94). For instance, miR-21 has been associated with osteoclast differentiation (94), while miR-29b promotes osteoblast differentiation and matrix mineralization (95).

#### 3.3.1 Assays for miRNA determination

MiRNAs are detectable in various body fluids such as blood (serum, plasma), and saliva, making them attractive non-invasive biomarkers (96). The main assays used for miRNA profiling include quantitative reverse transcription polymerase chain reaction (qRT-PCR), microarray, and next-generation sequencing (NGS) (97). However, each methodology has its own limitations (98), such as susceptibility to pre-analytical variation, low sensitivity and specificity, or high cost. Therefore, in addition to the need for further standardization, the selection of the appropriate analytical platform may be critically important.

#### 3.3.2 Clinical application

Particular attention from researchers is focused on uncovering the role of miRNAs in the diagnosis of osteoporosis. A large case-control study conducted by Shuai et al. (99) identified distinct circulating miRNA signatures, including miR-30c-2-3p, miR-199a-5p, miR-424-5p, miR-497-5p, miR-550a-5p, miR-654-5p, miR-663a, miR-877-3p, miR-1260b, miR-1299, capable of distinguishing individuals with osteoporosis from health and osteopenia, outperforming traditional bone turnover markers (BTM) (99). These miRNAs could provide additional value to dual-energy X-ray absorptiometry (DXA) for osteoporosis detection, independent of the participants’ age (99). Emerging diagnostic candidates for osteoporosis in postmenopausal women include miR-144-5p, miR-506-3p, miR-8068, and miR-6851-3p, which have shown superior diagnostic accuracy compared to traditional bone turnover markers (91). Notably, miR-144-5p exhibited a significant correlation with bone mineral density (BMD) at the lumbar spine, total hip, and femoral neck (91).

In a meta-analysis including 27 studies and a total of 2,263 participants with osteoporosis (100), Gao et al. reported a significant upregulation of miR-21-5p, miR-125b-5p, miR-483-5p, miR-133a, miR-422a, and miR-214-3p. Moreover, the profiling of miRNAs holds promising diagnostic and therapeutic implications in Paget’s bone disease (101), osteogenesis imperfecta (102), and rheumatoid arthritis (103). In oncological bone diseases, miRNAs also play crucial regulatory roles. Given their dual function as both oncogenes and tumor suppressors in bone sarcomas, Zoroddu et al. highlighted their potential use in the management and treatment of these cancers (104). Furthermore, miRNAs have been demonstrated to be useful in the diagnosis of multiple myeloma (105), in predicting the presence and burden of bone metastases in prostate cancer (106), and as potential therapeutic targets in bone metastases from hepatobiliary cancers (107).

The overview of the novel BTM was presented in **Table 1**.

**Table 1:** Overview of novel bone markers including sclerostin, DKK-1, RANKL/osteoprotegerin, PSTN, S1P, and microRNAs, regarding their origin, roles in bone metabolism, clinical relevance, and methods of determination. .

Biomolecule class	Marker	Bone tissue/ cellular origin	Function in bone	Assays	Clinical application
Proteins	Sclerostin	Osteocytes (mainly)	Inhibits Wnt/beta-catenin signaling, suppresses osteoblast activity, increases osteoclastogenesis by upregulating RANKL	ELISA (most common), MSD, currently unavailable automated CLIA	Potential biomarker for osteoporosis and fracture risk assessment; diagnostic adjunct in metabolic bone diseases (e.g., osteogenesis imperfecta, XLH, Paget’s disease); target of romosozumab therapy approved for osteoporosis treatment
	DKK-1	Osteocytes osteoblasts	Inhibits Wnt/β-catenin signaling by binding LRP5/6, suppressing osteoblast differentiation	ELISA	Potential biomarker and therapeutic target in osteoporosis, multiple myeloma, bone loss from glucocorticoids, rare bone disorders (e.g., osteogenesis imperfecta) and cancer-related bone disorders.
	RANKL and OPG	Osteocytes osteoblasts	RANKL stimulates osteoclastogenesis via RANK, while osteoprotegerin blocks this interaction, preventing osteoclast formation.	ELISA, but standardization and reproducibility is frequently not satisfactory; emerging multiplex electrochemical methods may be an alternative^1^	Potential biomarkers of osteoclast activity; elevated RANKL/OPG ratio linked to osteoporosis and low BMD. Limited diagnostic and monitoring utility without assay standardization.
	PSTN	Periosteum	Stimulates osteoblast differentiation and survival., promotes collagen cross-linking for strong bone formation, activates indirectly the Wnt/β-catenin pathway by downregulating sclerostin, supports fracture healing by aiding osteoblast function and matrix remodeling.	ELISA, automated CLIA	Potential biomarker of fracture risk in postmenopausal women and osteoporosis associated with primary hyperparathyroidism.
Lipid	S1P	Osteoclasts, osteoblasts, and their precursors, osteocytes	Regulates proliferation, apoptosis, and angiogenesis, coordinates osteoclast–osteoblast–endothelial cell crosstalk, recruits bone cell precursors	LC–MS/MS ELISA	Potential biomarker of fracture risk and early osteoporosis; therapeutic target via S1PR modulation (e.g., S1PR3 agonists ↑ bone formation, S1PR2 antagonists ↓ resorption); potential in bone regeneration and treatment of Paget’s disease
miRNAs	miRNA	osteoblasts, osteoclasts and their precursors, osteocytes	Post-transcriptional gene expression regulation, modulation of osteogenesis and osteoclastogenesis via Wnt/β-catenin, RANK/RANKL/OPG, BMP pathways, matrix mineralization	qRT-PCR, microarray, NGS	Potential biomarker and therapeutic role in osteoporosis, Paget’s disease, and osteogenesis imperfecta; potential therapeutic role in bone sarcomas; diagnostic biomarker in multiple myeloma; prediction and treatment of bone metastases (prostate and hepatobiliary cancer); bone healing

## 4 New endocrine aspects of the regulation of bone mineralization

Calcium and phosphate homeostasis, essential for skeletal remodeling and mineralization, is tightly regulated by three hormones: parathyroid hormone (PTH), vitamin D (vitD) and fibroblast growth factor 23 (FGF23). Its biological activity involves a complex interaction with multiple target organs including the kidneys, intestines, and parathyroid glands, as well as direct effects on bone cells. Other hormones influencing calcium and phosphate homeostasis are not discussed in this review.

### 4.1 Parathyroid hormone

PTH is an 84-amino acid peptide hormone synthesized in the parathyroid glands in response to changes in calcium levels (108). The amino-terminal fragment of PTH binds to the PTH1 receptor, which is expressed on osteocytes, osteoblasts, and bone lining cells, but not on osteoclasts (109). This interaction activates intracellular signaling cascades, primarily the cyclic adenosine monophosphate-protein kinase A (cAMP–PKA) pathway, and the phospholipase C– protein kinase C (PLC-PKC) pathway, which mediate its biological effects (110).

Advances in recent years in bone metabolism have significantly expanded understanding of PTH actions. Once regarded solely as a regulator of calcium-phosphate homeostasis, PTH is now recognized as a multifunctional hormone with direct effects on bone tissue through cellular and molecular mechanisms. Importantly, the effects of PTH depend on the mode of exposure—continuous hyperparathyroidism promotes bone resorption through indirect activation of osteoclasts, whereas intermittent administration exerts anabolic effects by stimulating the activity of osteoblasts and osteocytes. This principle is utilized in anabolic therapies for osteoporosis. These two forms of PTH administration trigger different gene regulations and signaling pathways. The catabolic effect of PTH is mediated through the promotion of osteoclastogenesis, achieved by upregulating RANKL expression in osteoblasts and osteocytes and downregulating OPG mRNA expression, which together shift the RANKL/OPG ratio in favor of bone resorption (110). PTH also increases the expression of monocyte chemoattractant protein-1 (MCP-1), thereby promoting the recruitment of pre-osteoclasts and enhancing RANKL-mediated osteoclastogenesis (111).

The anabolic effect of PTH is driven by multiple pathways that increase the number of osteoblasts, including the suppression of osteoblast apoptosis, the conversion of bone lining cells into active osteoblasts, the expansion of osteoblast precursors, and the stimulation of their differentiation into mature osteoblasts (112–114). Furthermore, PTH enhances Wnt signaling by inhibiting the expression of SOST, the gene encoding sclerostin, primarily in osteocytes, thereby promoting osteoblast activity and bone formation. The differential skeletal response to intermittent versus continuous PTH administration is not yet fully understood. One hypothesis suggests that the anti-apoptotic effect of PTH on osteoblasts is transient due to its influence on the proteolytic degradation of the runt-related transcription factor 2 (RUNX2). When RUNX2 levels decline below a critical threshold, PTH can no longer maintain its inhibitory effect on osteoblast apoptosis (115).

Clinical studies show that primary hyperparathyroidism is associated with elevated bone turnover markers, including formation markers such as OC, BALP and resorption markers, such as CTX-I (116). After parathyroidectomy, resorption markers decline rapidly, followed by a slower normalization of formation markers, accompanied by increases in bone mineral density, while serum sclerostin levels return to normal earlier than the other bone turnover markers (117, 118). In patients treated with teriparatide, formation markers, such as P1NP, rise quickly within days, and early changes in this marker correlate with subsequent gains in bone mineral density (119, 120).

#### 4.1.1 Analytical consideration

PTH is present in the circulation not only as the full-length active 84-amino acid peptide, but also as various fragments, predominantly derived from its C-terminal region, which contains the carboxyl-terminal part (121). These fragments, commonly referred to as C-terminal fragments, represent approximately 15%–30% of total PTH in healthy subjects and are either secreted directly by the parathyroid glands or generated through hepatic metabolism (122). They have a longer half-life than the full-length PTH and are eliminated from the bloodstream *via* the kidneys; therefore, they accumulate in patients with chronic kidney disease. Among these fragments, the 7–84 fragment is the most prevalent in circulation (123).

Currently, the primary method for determining PTH levels is immunoassay, which has undergone significant development over the years. Today, second- and third-generation automated sandwich-type immunoassay methods are commonly used. The second-generation assay, known as the intact PTH assay, uses two sets of antibodies targeting the C-terminal and N-terminal regions of the PTH molecule (124). However, the N-terminal antibody does not bind to the first four amino acids, which results in the detection of not only the biologically active full-length PTH (1-84PTH), but also C-terminal PTH fragments, most notably the 7–84 fragment (124). Third-generation PTH assays, also referred to as whole or bio-intact PTH assays, are designed to measure only the 1-84PTH. This is achieved through the use of an antibody directed at the first four amino acids of the N-terminal region, along with another targeting the C-terminal region, as in second-generation assay (123). Despite their higher specificity, third-generation assays may still detect posttranslationally modified forms of PTH, including those commonly overproduced in parathyroid carcinoma (123).

### 4.2 Vitamin D

Knowledge about the positive effect of vitD on bone mineralization and formation is well established. Guidelines unanimously recommend vitD supplementation to prevent nutritional rickets and support the attainment of peak bone mass during skeletal maturation, which is crucial for reducing the risk of osteoporotic fractures in later life (125). In adults and elderly, vitD prevents osteomalacia and reduces the risk of falls and fractures (126, 127). 1,25-(OH)_2_D, the hormonally active form of vitD, is a steroid hormone that exerts both direct and indirect effects on bone health. The indirect effect is due to the stimulation of calcium and phosphate absorption from the intestines, reabsorption in the kidneys, and inhibition of PTH secretion by decreased PTH gene expression (128). The direct effect is mediated by the presence of the vitD receptor (VDR) in osteoblasts (129). In studies using human osteoblasts, 1,25-(OH)_2_D has been shown to stimulate the differentiation of mesenchymal stromal cells into osteoblasts, promote osteoblast growth, and influence the mineralization process through the production of ALP-positive matrix vesicles (130). The regulation of these processes is mediated by the effect of 1,25-(OH)_2_D on the expression of genes involved in osteoblastogenesis and mineralization, including ALP, OC, and osteopontin (OPN) (131). On the other hand, studies using human bone cells have demonstrated that 1,25-(OH)_2_D enhances osteoclastogenesis by activating RANKL gene transcription in osteoblastic cells (132). This provides evidence that, similar to PTH, vitD is involved in both anabolic and catabolic effects on the skeleton.

Clinical studies on the impact of vitamin D supplementation on bone turnover markers yield inconsistent results. While the majority of studies report no significant changes (133, 134), some trials observed reductions in CTX-I (135) and PINP (136). In the study by Jorde et al., vitamin D supplementation that effectively suppressed high baseline PTH levels led to a marked decrease in PINP and CTX-I, along with an increase in serum sclerostin, indicating reduced bone turnover through PTH suppression (136). The variability of findings across studies may be related to differences in dosage, treatment duration, baseline vitamin D and PTH status, or differences in calcium intake.

The initial metabolites used to synthesize hormonally active 1,25-(OH)_2_D are: cholecalciferol (vitD_3_) and ergocalciferol (vitD_2_). Subsequently, as the result of 25-hydroxylation by cytochrome P450 family 2 subfamily R member 1 (CYP2R1) mainly in the liver, 25-(OH)D_2_ and 25-(OH)D_3_ are formed, then during 1-alpha-hydroxylation by cytochrome P450 family 27 subfamily B member 1 (CYP27B1), 1,25-(OH)_2_D_2_ and 1,25-(OH)_2_D_3_ are synthesized, respectively. The inactivation of 1,25-(OH)_2_D_3_ and 25-(OH)D_3_ is mediated by the enzyme 24-hydroxylase (cytochrome P450 family 24 subfamily a member 1 (CYP24A1)), which plays a crucial role in the vitD catabolism. The direct products of the CYP24A1 reaction are 24,25-(OH)_2_D_3_ and 1,24,25-(OH)_2_D_3_, which are further converted to calcitroic acid destined for biliary excretion. However, recent studies suggest that 24,25-(OH)_2_D_3_ is not simply a degradation product of vitD metabolism, but a metabolite that may play a role in bone formation. *In vivo* animal models, its role in fracture healing has been demonstrated (137). Furthermore, studies using mesenchymal stem cell cultures have shown that 24,25-(OH)_2_D_3_ is involved in their differentiation into osteoblasts (138, 139).

#### 4.2.1 Analytical consideration

Currently, the LC-MS/MS technique enables the reliable determination of a vitD metabolite panel metabolites simultaneously, including 24,25-(OH)_2_D and 3-epi-25-(OH)D, offering a new perspective on the assessment of vitD status and the potential for rapid detection of vitD metabolism disorders. The evaluation of vitD status is typically based on the measurement of the total serum concentration of 25-(OH)D. It results from the relatively stable expression of the 25-hydroxylase gene, indicating that the concentration of 25-(OH)D is primarily depended by the availability of its substrate. However, recent studies indicate that the ratio of 24,25-(OH)_2_D to 25-(OH)D multiplied by 100, known as the vitamin D metabolite ratio (VMR), may serve as a more reliable marker of vitD status. There are several points that support this hypothesis.

VitD, like other steroid hormones, is highly lipophilic and therefore needs a carrier protein in the serum for delivery to target tissues. Approximately 85%-90% of 25-(OH)D is bound to vitD binding protein (VDBP), which is the non-bioavailable fraction (140). However, studies report significant individual differences in the concentration of binding proteins. In the study by Powe et al., black Americans had lower levels of 25-(OH)D and VDBP, resulting in similar concentrations of estimated bioavailable 25-(OH)D compared to white Americans (141). Genetic polymorphisms in VDBP, health status, pregnancy, and medications that affect VDBP concentrations may contribute to the variability in 25-(OH)D levels. Therefore, low 25-(OH)D levels may not necessarily reflect true vitD deficiency. Many individuals with low 25-(OH)D levels do not exhibit clinical symptoms of deficiency or elevated PTH levels. Black Americans in the above study had a higher bone mineral density and lower risk of fractures than white Americans, despite lower 25-(OH)D concentrations (141). Since VDBP affects both the numerator and denominator of the VMR ratio, the final VMR value is likely not affected by its influence. Dugar A. et al. measured the concentrations of 25-(OH)D, 1,25-(OH)_2_D, 24,25-(OH)_2_D_3_, and VDBP in patients before and after therapeutic plasma exchange (TPE), a procedure that removes plasma, including VDBP (142). A significant decrease in the concentrations of VDBP and the determined vitD metabolites was observed, but no significant change in VMR was detected (142). Moreover, including the metabolite 24,25-(OH)_2_D in vitD status assessment provides more dynamic and functional information on vitD deficiency. In a recent study with 1200 Belgian children, it was shown that, despite having the same 25-(OH)D concentration, some individuals had already begun to catabolize 25-(OH)D, showing measurable levels of 24,25-(OH)_2_D, while others did not (143). This suggests the possibility of a personalized threshold for metabolism (143). In the study by Hermann et al., low VMR (< 4%) was found to be associated with significantly higher PTH levels, increased bone metabolism, and elevated all-cause mortality, regardless of serum 25-(OH)D concentration (144).

An additional clinical use of the VMR is its role as a biomarker for identifying loss-of-function mutations in the CYP24A1 gene. The loss of 24-hydroxylase function can result in severe hypercalcemia in infants or milder forms of hypercalcemia in adults, depending on the specific pathogenic variant (PV) (145). The measurement of 24,25-(OH)_2_D_3_ is crucial for distinguishing patients with CYP24A1 mutations from those with other causes of PTH-independent hypercalcemia, including intoxication. In cases of suspected CYP24A1 mutations, the VMR is typically expressed oppositely compared to vitD status assessment, with 25-(OH)D as the numerator and 24,25-(OH)_2_D_3_ as the denominator. A VMR ratio exceeding 80 (a reference range of 5 to 25) indicates a genetic defect in the CYP24A1 gene (146).

### 4.3 Fibroblast growth factor 23

FGF23 is a 32 kDa glycoprotein composed of 251 amino acids, classified within the FGF family of signaling molecules (147). It is a phosphaturic hormone primarily secreted by osteocytes and osteoblasts. FGF23 expression is increased by calcitriol, PTH, and high dietary phosphate intake. It acts on the FGF23 receptor, which is mainly expressed in the proximal tubules of the kidney and parathyroid gland cells. The FGF23 receptor is a complex consisting of a tyrosine kinase FGF receptor and the α-Klotho coreceptor. α-Klotho is a transmembrane protein predominantly expressed in the distal tubules of the kidneys. α-Klotho associates with FGF receptors and functions as a cofactor for FGF23, facilitating its binding to target receptors and activation of downstream signaling pathways.

The main effect of FGF23 is exerted in the renal proximal tubule, where it inhibits phosphate reabsorption and suppresses calcitriol production. By downregulating sodium-phosphate cotransporters NaPi2a and NaPi2c, FGF-23 promotes phosphate excretion in urine. It also reduces the renal conversion of 25-(OH)D to 1,25-(OH)_2_D, leading to decreased calcitriol levels and, consequently, reduced intestinal absorption of calcium and phosphate. These combined actions result in a decrease in serum phosphate levels. Additionally, FGF23 suppresses PTH synthesis and secretion by acting directly on the parathyroid glands.

The FGF23/Klotho axis has a well-established role in the pathogenesis of chronic kidney disease–mineral and bone disorder (CKD-MBD), with FGF23 levels rising in the early stages of CKD (148). However, recent findings highlight broader involvement of FGF23 in the physiology of bone remodeling (149), suggesting its potential utility as a bone turnover marker. FGF23 may affect bone mineralization and osteoblasts by regulating key markers such as OPN and alkaline phosphatase (150). FGF23 may also influence bone resorption by modulating osteoclast development (151), although further research is needed to clarify its direct effects and the specific role in this process.

#### 4.3.1 Analytical consideration

FGF23 can be detected using various immunoassay methods in serum or plasma. The majority of commercially available assays detect intact FGF23 (iFGF23), but there are also methods available to measure the C-terminal fragment (cFGF23). iFGF23 detects the active hormone but is less stable due to preanalytical degradation and diurnal variation (152). cFGF23 assays offer greater stability and lower biological variability, but they also detect inactive fragments that may exert counter-regulatory effects on the active hormone, potentially complicating interpretation in studies focused on FGF23 biological activity (153). No international standard exists for FGF23 assays, and available comparisons reveal significant variability and lack of harmonization, especially among intact FGF23 tests (154), while C-terminal assay comparisons are currently unavailable. FGF23 serum concentrations are significantly higher in females than in males and remain relatively stable throughout adulthood, with a slight increase in old age (155).

Elevated FGF23 has been linked to postmenopausal osteoporosis, with several studies showing a negative correlation between serum FGF23 levels and BMD in postmenopausal women (156, 157). By comparison, evidence regarding osteoporosis in aging men is less consistent, with studies reporting either weak or no significant associations between FGF23 levels and BMD (158, 159). Currently, available evidence remains insufficient to support the use of serum FGF23 as a reliable marker in the evaluation of osteoporosis in the elderly. Given the role of FGF23 in the pathogenesis of chronic kidney disease, the protein has been investigated as a potential biomarker of osteoporosis in patients with CKD or end-stage renal disease (ESRD). While elevated FGF23 has been identified as a fracture risk factor in CKD patients, no studies have demonstrated a negative correlation between FGF23 levels and bone mineral density (160, 161).

## 5 New nomenclature of biochemical indices of bone status

According to the recently published recommendations by the Joint International Osteoporosis Foundation (IOF) Working Group and the International Federation of Clinical Chemistry and Laboratory Medicine (IFCC) Committee on Bone Metabolism, all biochemical indicators reflecting skeletal metabolism are now collectively referred to as Bone Status Indices (BSIs) (162). This unified nomenclature replaces the traditional concept of BTM and expands it to include structural markers, bone cell enzymes, as well as hormonal and regulatory components. Structural BSIs comprise indices derived from type I collagen metabolism, including PINP, NTX-I, CTX-I and their variants (162). Enzymatic groups are represented by BALP, TRACP5b, and cathepsin K (CTSK), while regulatory components encompass endocrine and paracrine mediators such as PTH, vitD metabolites, FGF23, OC, Wnt/β-catenin inhibitors (sclerostin, DKK-1), TNF superfamily members (RANKL, OPG), and factors involved in cell migration and adhesion, such as PSTN, OPN and secreted protein acidic and rich in cysteine (SPARC) (162). This classification emphasizes the integrative nature of bone metabolism and facilitates standardization of terminology, abbreviations, and measurement units for BSIs, supporting consistent interpretation of biochemical bone status.

## 6 “Omics” approaches to assessment of bone turnover

In recent years, there has been growing interest in the “omics” techniques. Among these techniques, metabolomics allows to analyze entire panels of low weight compounds (< 1500 Da) produced by structures of a selected magnitude: from single cells to entire organisms (163). This approach offers a unique insight into metabolic processes and often enables us to uncover new biomarkers, with potential clinical relevance. The development of metabolomics (targeted and untargeted) would not have been possible without the analytical advancement: mainly mass-spectrometry based methods and nuclear magnetic resonance (NMR) spectroscopy (164).

Given the complex and not fully understood process of bone remodeling and its disturbances, the application of metabolomics is studied intensively in the context of bone formation and resorption (165, 166). In the study of Bellissimo et al., bone formation biomarker P1NP was associated with multiple metabolic pathways including several amino acids (alanine, beta-alanine, arginine, aspartate, glutamate and proline, the latter being one of main components of collagen type I), vitamin C (crucial for the procollagen hydroxylation and secretion), B vitamins (i.a., thiamine and niacin, precursors of coenzymes involved in catabolic reactions), tricarboxylic acid (TCA) cycle, and pyruvate metabolism (167, 168). In contrast, the serum concentration of bone resorption biomarker CTX was associated with fatty acids and lipid metabolism pathways (167). The results correspond with the observation that actively resorbing osteoclasts are rich in mitochondria ensuring high capacity of beta-oxidation of the fatty acids and osteoclasts may be mainly supported by energy-dense lipid, rather than carbohydrate catabolism (167, 169). Another metabolomic study led by Hartley et al. on individuals with high bone mass (Z-score ≥+3.2), measuring absolute concentrations of more than 150 metabolic traits using NMR spectroscopy, identified an association between beta-CTX concentration and plasma citrate – first product of TCA cycle (170). However, cellular metabolism of the soft tissues is not the main source of citrate in the bloodstream – around 80% of citrate is bound in the bones with hydroxyapatite and enters circulation after the bone is resorbed (171). Alongside with the assessment of low-weight metabolic compounds of bone turnover, novel analytical techniques also allow to determine entire panels of proteins. In a proteomic study, led by Bhattacharyya et al., the use of surface enhanced laser desorption ionization (SELDI) time-of-flight mass spectrometry (TOF-MS) allowed to determine a proteomic profile discriminating postmenopausal patients with high and low/normal bone turnover (172). Furthermore, four of the discriminatory peaks were identified as fragments of interalpha-trypsin-inhibitor heavy chain H4 precursor (ITIH4), kallikrein-sensitive glycoprotein present in the blood, which may serve as a biomarker of increased osteoclast activity (172).

In the context of bone remodeling assessment, the application of metabolomics and other “omics” techniques provides a comprehensive, dynamic, and informative view. It may also clarify the link about cellular metabolism and bone remodeling, and eventually support the personalized choice of therapy and monitoring. However, metabolomics is remarkably limited by biological variability of determined panels, platform-dependent coverage, large amounts of generated data, lack of standardization, which hinder reproducibility and clinical translation. In addition, many associations between determined compounds and bone remodeling remain correlative rather than causal, underlining the need for validation and integration with well-established BTM.

## 7 Conclusions

The understanding of bone remodeling has advanced beyond the scope of traditional BTM, which provide only limited specificity and do not reflect osteocyte activity. Emerging biomarkers, including proteins, lipids, miRNAs, and the application of “omics” techniques, offer deeper insight into the cellular and molecular mechanisms regulating skeletal integrity and are potential therapeutic targets. Simultaneously, hormonal modulators such as PTH, vitD, and FGF23 coordinate this process, influencing not only systemic mineral balance but also local bone cell activity, thereby integrating mineralization with overall remodeling dynamics. However, novel BTM cannot be viewed as substitutes for classical BTM but rather as complementary tools, and further studies are required to clarify their role in specific clinical settings.
